# Bipolar charge trapping for absorption enhancement in a graphene-based ultrathin dual-band terahertz biosensor

**DOI:** 10.1039/d1na00388g

**Published:** 2021-08-30

**Authors:** Gaurav Varshney, Pushpa Giri

**Affiliations:** ECE Department, National Institute of Technology Patna India-800005 gaurav.ec@nitp.ac.in

## Abstract

Surface plasmons generated at the graphene dielectric interface can be altered by trapping the electric charge. A technique is implemented for trapping the bipolar electric charge on the graphene surface and arranged in a desired way to enhance the performance of a monolayer graphene metamaterial based tunable, ultrathin, dual narrow band terahertz (THz) absorber. A monolayer graphene sheet placed on the dielectric substrate can provide dual-band resonance by utilizing the surface plasmons of the fundamental and third order mode index and an absorption of more than 99% and 50% can be obtained in the lower and upper band, respectively. The absorption is further enhanced to the level of perfect-absorption by utilizing the charge trapping mechanism on the graphene and generating bipolar charged nodes to create higher order surface plasmons. The multiple interference and reflection theory proves that the destructive interference in the dielectric layer is the cause of perfect absorption. The applied technique in the dual-band absorber configuration provides a tunable response which remains insensitive to the polarization and incident angle of the electromagnetic wave. The proposed perfect absorber can be utilized as a biosensor for refractive index sensing and the detection of glucose in water and the malaria virus in blood. It can provide an ultrahigh sensitivity of 14.88 THz RIU^−1^ with FOM as 53.09 RIU^−1^ with the variation in the chemical potential of graphene and 12.7 THz RIU^−1^ and FOM as 41.1 RIU^−1^ during glucose detection in water.

## Abstract 2

Surface plasmons generated at the graphene dielectric interface can be altered by trapping the electric charge.

## Graphical abstract

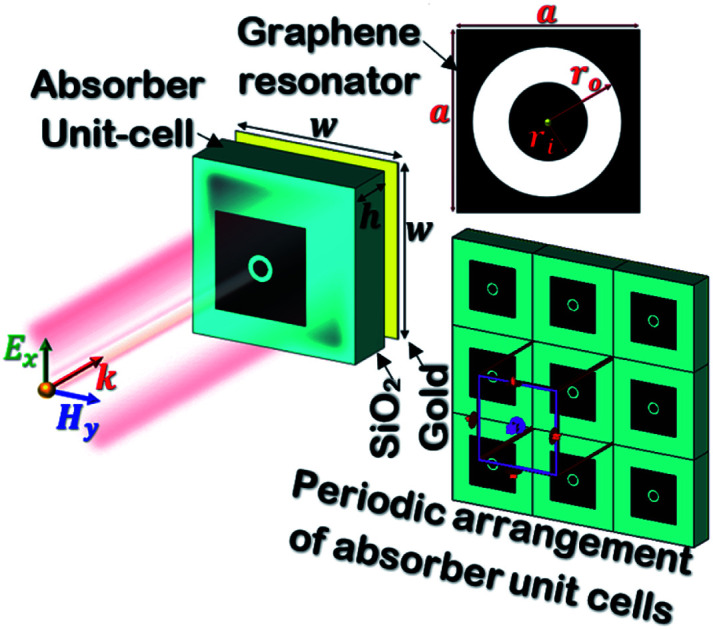

## Introduction

Artificial materials with specific ranges of electrical parameters like permittivity and permeability have revolutionized high frequency applications.^1^ Such artificial materials are formed with some spatial sequencing of the unit cell and are called metamaterial (MM). The electromagnetic properties of such materials are not found in natural materials. Their extraordinary properties made the implementation of cloaks,^2,3^ an invisible carpet^4^ and a perfect lens^5^ possible. The current research trends are moving in the direction of implementing terahertz (THZ) devices like antennas,^6^ sensors,^7–9^ filters^10^ and absorbers.^11^ The purpose of implementing THz absorbers is enabling the technology in spectroscopy,^12^ detection and sensing^13^ and many more applications.^14^ Recently, the implementation of ultrathin,^15^ ultrasensitive^8,13,16,17^ absorption based sensors/biosensors with a narrow^17,18^ and ultra-narrowband^19,20^ response is being investigated. Recent implementations discuss the development of narrowband sensors with multi-layered media with complex structures with a large thickness.^17,20,21^ Another challenge in the implementation of sensors is making the response independent of the incident angle and polarization of the incident electromagnetic wave.^7,11^ This feature enables the sensor to be used in dynamic sensing systems which can be utilized in handheld devices.^22^ All these features of absorption-based sensors can be obtained by utilizing MM in THz implementations. In MM sensors, the localized surface plasmon resonance created due to free electrons at the highly conductive material and dielectric interface changes with the variation in the surroundings like the refractive index of the medium.^23–25^ This leads to a change in the spectral behaviour of the MM absorber and thus it can be utilized as a sensor.^26^ Specifically, biosensing applications require a narrowband sensor with perfect absorbance which can be possible with MM cell implementations due to the strong electric and magnetic resonance caused in the periodic structure.^20,27–29^ In the sequence of implementing the MM absorber, graphene has been introduced to obtain tunability and controllability in the frequency spectrum.^8,30^ The electrical behaviour of graphene for varying chemical doping and external electrostatic or magnetostatic field allows the impedance matching to free space which remains the primary requirement in mitigating the back reflections in the absorbers.^6^ The excitation of graphene surface plasmons (GSPs) causes a strong field confinement which results in a perfect absorption spectrum.^8^ In fact, the nanoscale fabrication can be miniaturized by the usage of graphene due to the strongly confined wave vector over the propagation in free space, which may help in providing ultrathin geometries.^31–33^

This work reports a MM absorber exploiting a graphene patch and providing dual-band resonance with perfect absorption. The structure offers an ultrathin geometry at the THz spectrum with a thickness of around thirty times scaled down of the free space wavelength. The monolayer graphene patch provides an absorption of more than 99% and 50% in the lower and upper band, respectively. A circular slot is inserted in the graphene sheet which traps the electric charge and hence bipolar charged nodes are created in the graphene sheet which enhances the impedance matching in the upper band and hence the absorption to more than 99%. The lower and upper band absorption peaks are the result of fundamental and higher order plasmonic resonance, respectively. The interference theory proves that absorption occurs due to multiple reflections caused in the dielectric substrate being utilized between the slotted graphene patch and metal layer.^34–36^ The frequency behaviour of the absorption spectra changes with the variation in the refractive index of the surroundings when a plane wave impinges on the graphene surface. Thus, the proposed narrow band absorber can be utilized in potential biosensing applications like in the detection of glucose in water and malaria with good performance indices like high sensitivity, figure-of-merits and quality factors. The absorber response remains independent of polarization and the incident angle of the electromagnetic wave shows the high degree of symmetry of the structure.^37^ The electrical bias voltage applied on graphene can provide the tunability and controllability of the absorption peaks. Moreover, the implemented absorbing unit behaves as a perfect wideband reflector in between the absorption peaks and thus can be utilized in the frequency selective THz sources. Generally, multiple or dual-band resonances are obtained in complex multi-layered structures.^11,37–40^ In comparison, the proposed ultrathin dual-band absorber can offer a geometry with less complexity. The experimental realization of metal-based nanoscale devices has already grown at a great level.^24,25,28,29,41–43^ However, the fabrication of graphene metamaterial based absorbers is still a challenging task and thus simple geometries are being implemented and investigated.^30,44,45^ This research work advances the literature and paves the way to implementing graphene metamaterial-based absorbers. The reported research work is mainly categorized by two outcomes; (i) implementation of a technique for alteration of surface plasmons on graphene and trapping the electric charge for the creation of bipolar charged nodes. This establishes the equality of the mode index of surface plasmons at the graphene-dielectric and graphene–air interface, which results in perfect absorption at the frequency of higher order resonance and (ii) the utilization of the proposed ultrathin, ultra-sensitive, tunable dual narrowband absorber as a biosensor for refractive index sensing and the detection of glucose in water and the malaria virus in blood.

## Absorber design

**Fig. 1:**
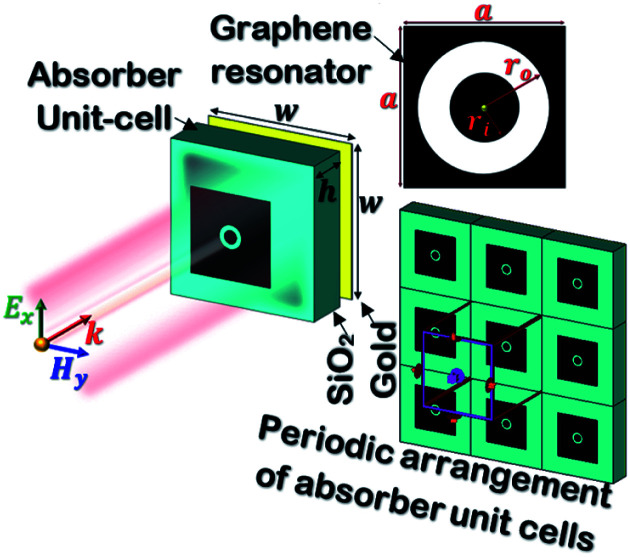
The absorber unit cell, graphene resonator and its periodic arrangement (*a* = 5, *r*_o_ = 0.6, *r*_i_ = 0.4, *w* = 9, *h* = 2.5 μm, *μ*_c_ = 0.4 eV, *T* = 300 K, *τ* = 0.1 ps and graphene thickness, *t*_g_ = 0.34 nm).

Fig. 1 shows the geometry of the proposed absorber. This contains a silicon dioxide (SiO_2_) substrate with relative permittivity, *ε*_s_ = 2.25 and height *h*. A square-shaped graphene resonator of edge *a* is placed at the top of the substrate. This graphene resonator contains an annular circular slot of outer and inner radius *r*_o_ and *r*_i_, respectively. This whole structure is placed at the top of the metallic sheet. In this structure, gold material is used as the metal plane or back reflector. The thickness of the metallic plane is kept such that it remains more than skin depth for proper reflection of the incident wave. The numerical analysis of the absorber is performed with the considerations of the periodic arrangement of the unit cells having periodicity *w* along the *x* and *y*-axis and Floquet port boundaries. The simulation is performed with 100 cells per wavelength and the total number of tetrahedrons as 733 917 along with the adaptive meshing.^46^ The incident electromagnetic wave from medium-2 (air) on the graphene sheet creates the p-polarized surface plasmons (SPs) with decaying electric field strength over the transverse distance.^31,47^ The dispersion relation of p-polarized surface plasmons (SPs) in a graphene patch placed on a substrate can be obtained from Fresnel's relations as given in eqn (1).^48^1
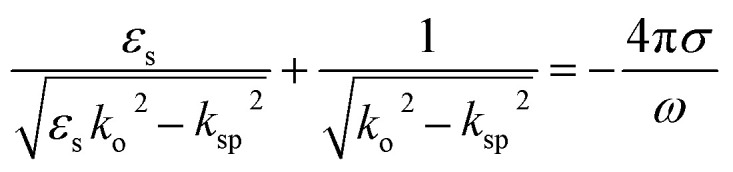


Here, 
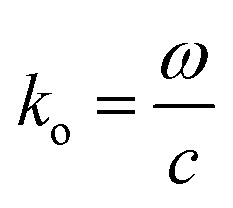
, is the free space wave number, *ω* is the angular frequency and *c* is the speed of light. The electrostatic limits can be applied for obtaining the plasmons vectors as 
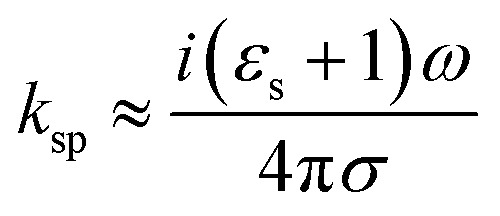
 under the condition *k*_o_ ≪ |*k*_sp_|. Now, Drude's conductivity^47^ expression modifies the surface wave vector as 
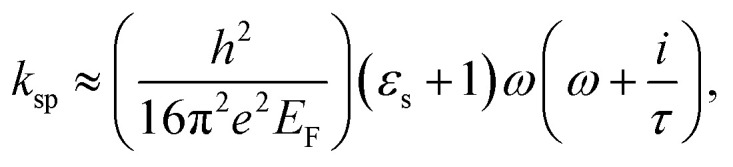
 with *τ* as the relaxation time of graphene. This quadratic expression confirms the presence of 2-dimensional electron gas on the graphene-dielectric interface. The degree of confinement can be defined in terms of the surface plasmon wavelength *λ*_sp_ in respect to the free space wavelength *λ*_o_ as 
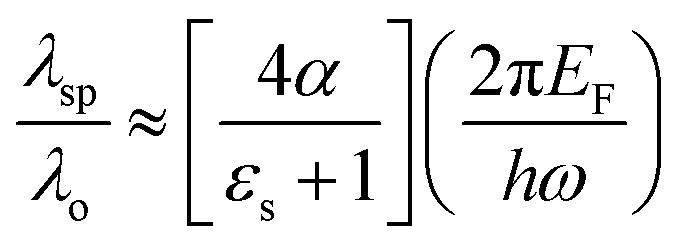
. Here, *α* ≈ 1/137 which is the fine-structure constant, *h* is the Plank's constant and *E*_F_ is the fermi-energy.^31,47^ The edge length of the graphene patch is selected as *a* ≈ *λ*_sp_/2 at frequency 4.4 THz for the value of graphene chemical potential as *μ*_c_ ≈ 0.3 eV, relaxation time *τ* = 0.1 ps at temperature *T* = 300 K. The minimum thickness of the substrate that provides the perfect absorption remains around *h* ≈ *λ*_sp_/4.

The accurate fabrication methods of the micro/nanoscale graphene-based absorbers are currently being investigated. The fabrication of metal-based metamaterial implementation has already been reported frequently in the literature.^24,25,28,29,41–43^ The fabrication of graphene-based absorbers is still a challenging task. There have been very few reports on the experimental validation of graphene-based absorbers.^30,44,45^ A method of fabrication of graphene-based absorbers is reported quoting the chemical vapour deposition (CVD) process and transfer approach.^30^ Monolayer graphene can be grown using the CVD process and then transferred on the SiO_2_ substrate. After that, the graphene sheet can be patterned using e-beam lithography. A detailed process of fabrication of the patterned graphene sheet is demonstrated in ref. 45.

## Results and analysis

**Fig. 2:**
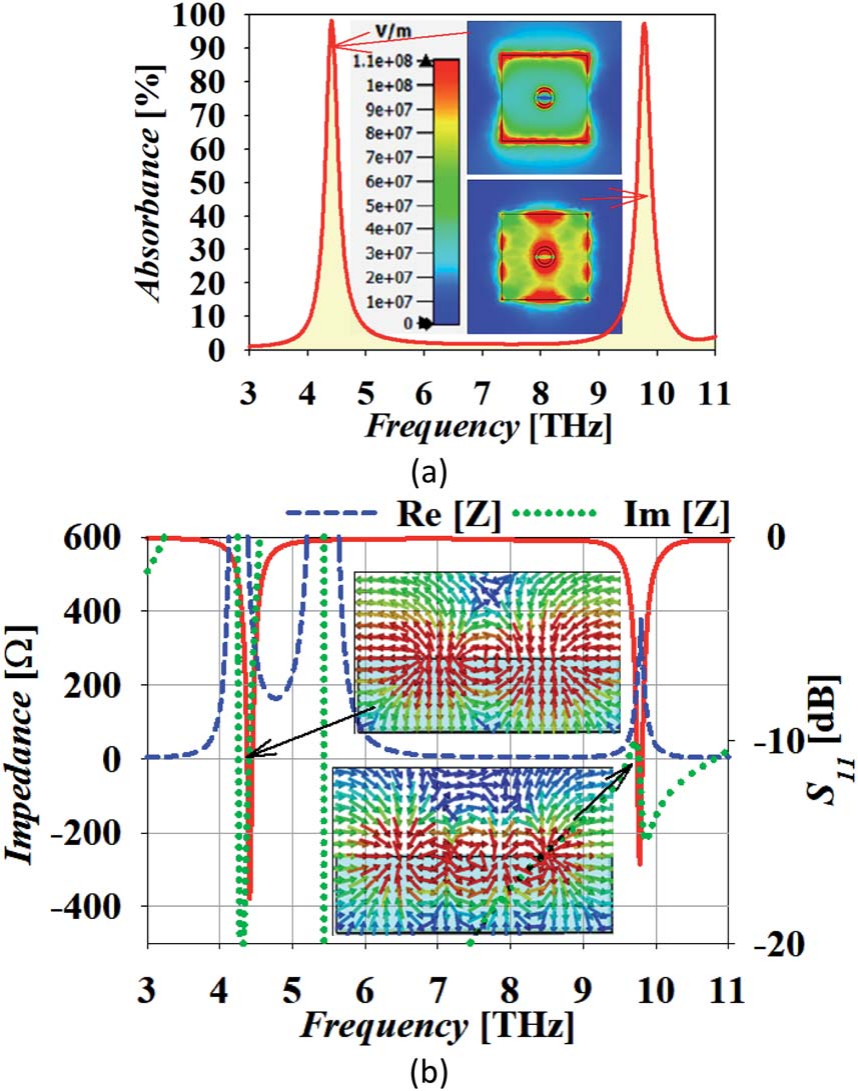
(a) The absorption spectrum and contour plot of the absolute electric field in the *xy*-plane and (b) the impedance plot and vectored electric field (*E*_*x*_- magnitude) in the *xz*-plane of unit-cell at *y* = 0 μm.

A unit cell is analyzed with the plane wave incident at normal to the arrangement of electric and magnetic fields along the *x*- and *y*-axis, respectively. The floquet ports are applied with the air spacing from the top and bottom surface of the absorber. Fig. 2 shows the absorber response. The proposed absorber provides an absorption peak of more than 99% at the resonant frequencies 4.44 and 9.864 THz. It is worth noting that the frequency response of the proposed absorber remains spurious free over the frequency spectrum of observation as well as behaving as a perfect reflector in the mid frequency ranges other than the absorption spectra is obtained. The absolute *E*-field distribution shown in Fig. 2 on the absorbing unit confirms that fundamental and higher order SP resonance is created by the graphene patch at the frequency of the lower and upper band peak, respectively. The field distribution shows one maximum at the edges of the graphene patch at the peak frequency of the lower band. There are three field maxima at the centre of the graphene patch at the peak frequency of the upper band. The magnitude of the vectored E-field at frequency 4.44 THz in the lower band and 9.86 THz in the upper band corresponds to the mode with index *m* = 1 and 3, respectively.^31^ This field distribution is reported for transverse electric (TE) polarization. The field distribution for transverse magnetic (TM) polarization becomes orthogonal phase shifted with similar characteristics and mode indices. The reflected wave from the graphene resonator must be as minimum as possible. This is confirmed by drawing the impedance plot as shown in Fig. 2(b). The real part of the impedance matches the free space impedance with its zero-imaginary part at the resonant frequencies confirming the perfect impedance matching.

## Absorber evolution

**Fig. 3:**
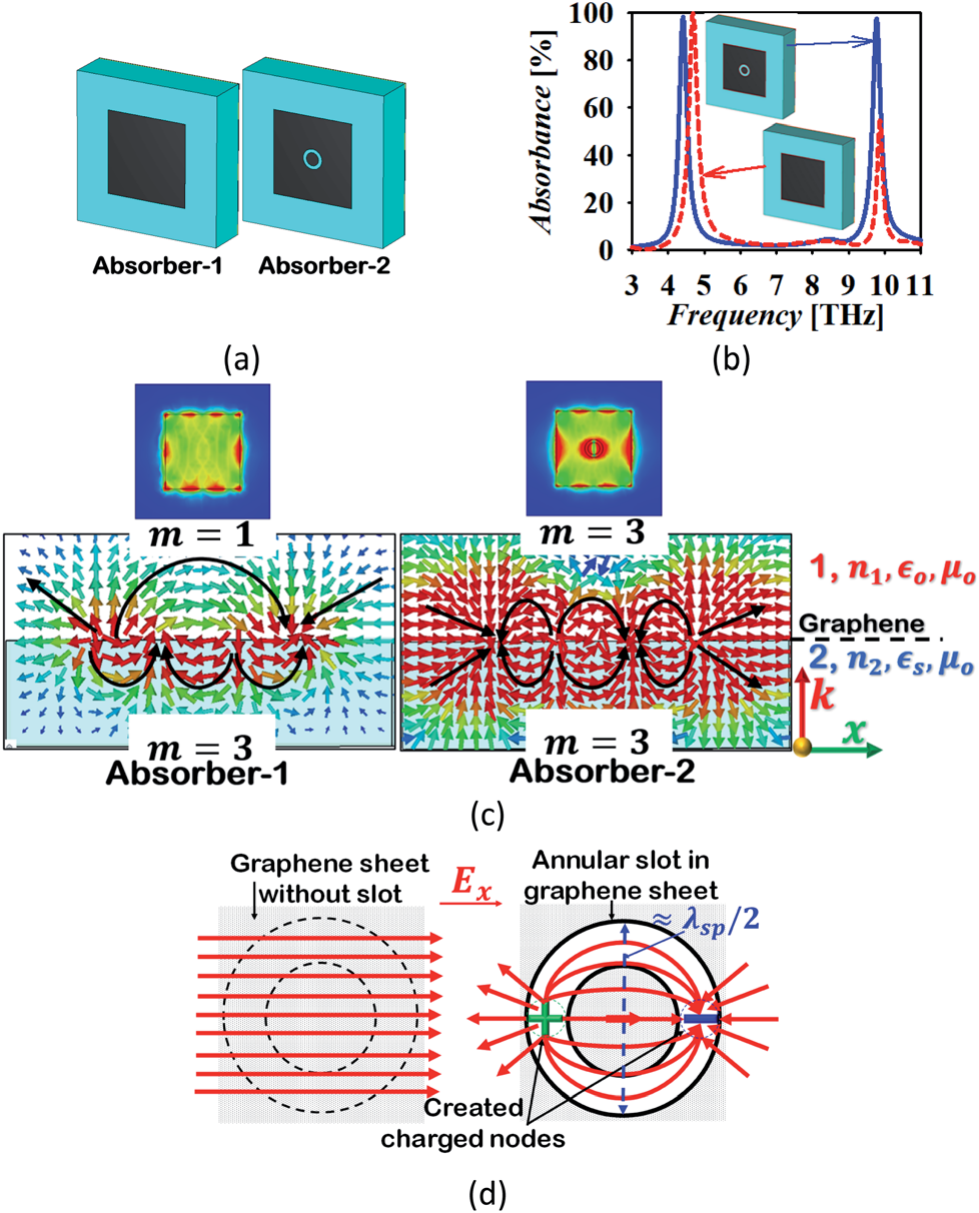
(a) The absorber evolution, (b) the response, (c) the computed absolute and vectored modal field distribution (*E*_*x*_-magnitude) in the *xz*-plane at *y* = 0 μm and the surface plasmons on the graphene-dielectric interface at the frequency of higher order resonance showing the creation of bipolar charged nodes in the graphene sheet by inserting an annular slot and (d) the charge trapping mechanism for the creation of bipolar charged nodes by inserting the slot in the graphene sheet.

Two absorber structure configurations have been numerically analysed to understand the operation of the proposed dual-band absorber. The first structure (absorber-1) contains a graphene based rectangular resonator placed on the substrate and makes an interface with air providing two resonances at around 4.68 and 9.84 THz. This absorber provides an absorption peak with an absorptivity of more than 99% and 50% at the frequency of the first and second resonance, respectively. Absorber-1 offers a poor impedance matching in the upper band which results in the low value of absorption. For the enhancement of the absorption at a higher frequency, a circular annular slot is inserted in the rectangular graphene resonator. As a result, the absorption is enhanced to the level of more than 99% in the upper band, as shown in Fig. 3(b). The computed modal field distribution in the absorber structures is analysed at the resonant frequencies and is illustrated in Fig. 3(c). The structure of absorber-1 provides an unequal field confinement at the frequency of higher order resonance at the graphene interface with different mediums. It can be noticed in the field distribution that there are four and two nodes of surface charge polarity in medium-1 and 2, respectively. Correspondingly, the mode number in medium-1 and 2 correlates with *m* = 1 and 3, respectively. This discontinuity results in a low value of absorption at the frequency of higher order resonance.^31^ It can be predicted in the field distribution that absorption can be enhanced at the frequency of the higher order resonance if an equal charge distribution is obtained along the graphene interfaces. This can be done by creating the electric charge storage geometry in the graphene sheet which has dimensions equivalent to the one half-wave variation of the surface plasmons wavelength. Also, the inserted geometry must be symmetrical to maintain the insensitivity to the polarization angle of the incident electromagnetic wave. The best way is the insertion of an annular circular slot of diameter equivalent to *λ*_sp_/2 at the frequency of higher order resonance. The creation of a slot provides the capacitive effect with the capability of the storage of electric charge with opposite polarity along the orientation of electric field vectors.^49^ Thus, two extra nodes are created in the centre of the graphene sheet at both the ends of *λ*_sp_/2 at a frequency around 9.85 THz which helps in maintaining the mode index as equal in both the sides of the graphene sheet. The creation of the charged nodes and their effect is illustrated in Fig. 3(d). It shows that the annular slot offers the capacitive storage of charge with opposite polarity, which establishes the equal mode index at the graphene–air interface. Consequently, the field is strongly confined and absorption is enhanced. Furthermore, the mode index at the graphene–air and graphene–dielectric interface remains similar at the frequency of fundamental mode. Moreover, the resonant frequency of the fundamental and higher order mode shifts after insertion of the slot to 4.44 and 9.86 THz, respectively.

## Validation through interference theory

**Fig. 4:**
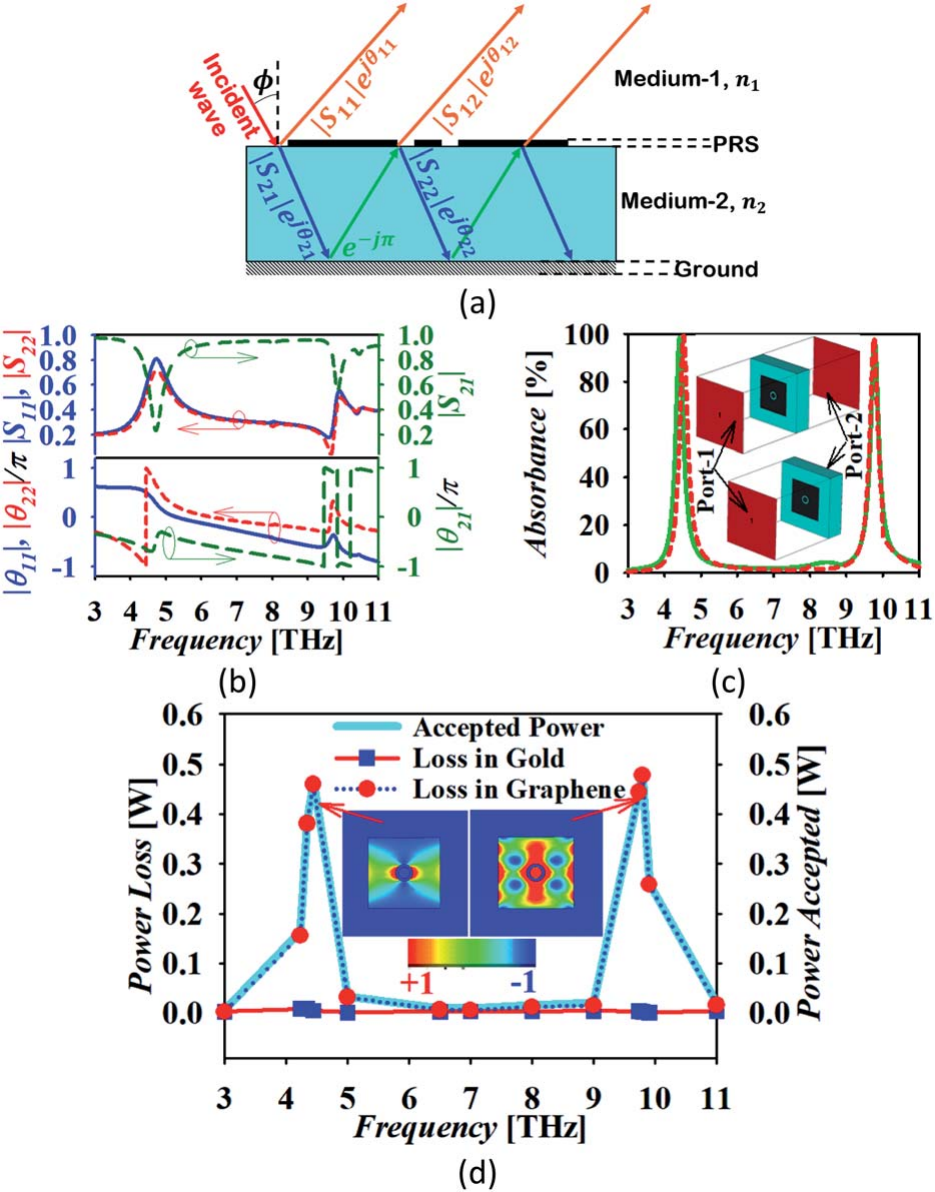
(a) The multiple interference and reflection theory model, (b) the magnitude and phase of reflection and transmission coefficient for the decoupled system, (c) the simulated and calculated absorption plot for the coupled (solid line) and decoupled system (dotted line) at normal incidence with the representation of coupled and decoupled systems and (d) the power profile in the absorbing unit-cell in a coupled system.

The operation of the proposed absorber is verified with the help of the interference theory.^34–36^Fig. 4(a) shows the multiple interference and reflection model of the proposed absorber. The theory of reflection from a multi-layered media is applied with the consideration of coupled and decoupled systems for analysis. In the coupled system, a ground plane is applied and absorption is calculated based on the obtained reflection and transmission coefficient with the consideration of two ports. In the decoupled system, the metal layer being used as the ground is removed from the structure and port-2 is applied just at the dielectric–air interference as shown in Fig. 4(c). There are two mediums 1 and 2 with a refractive index of *n*_1_ and *n*_2_, respectively. Here, the graphene patch is considered as a partially reflecting surface (PRS). A fraction of the incident wave from medium-1 is transmitted into medium-2 and reflected back to medium-1 with the magnitude |*S*_21_| and |*S*_11_| and phase e_21_^*jθ*^ and e_11_^*jθ*^, respectively. The metallic surface at the back provides the fully reflected wave with the addition of the phase factor e^−*j*π^. This wave is again reflected back in medium-2 and transmitted in medium-1 with the expression as |*S*_22_|e_22_^*jθ*^ and |*S*_12_|e_12_^*jθ*^. The magnitude and phase of all the transmitted and reflected waves are extracted from the full wave simulation using the CST microwave studio in the decoupled system, which is drawn in Fig. 4(b). The magnitude and phase plot of the transmitted and reflected wave at the interface shows that there are dips in the transmittance with the peak of the reflection at the resonant frequencies. This confirms the creation of resonance due to the slotted graphene patch at two frequencies. Correspondingly, the phase of the waves shows its variation at the resonant frequencies accordingly satisfying the necessary and sufficient conditions.^34–36^ The total reflection coefficient *R*(*ω*) in medium-1 is then calculated using eqn (2).^35^2
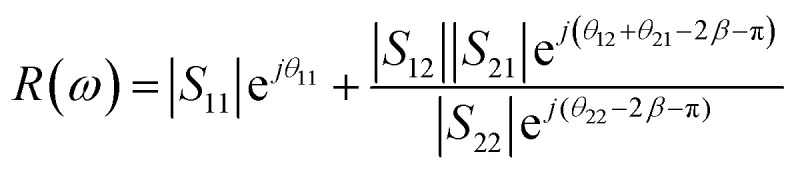


Here, *β* = *kd* is the propagation constant with *k* as the wave number in medium-2 and 
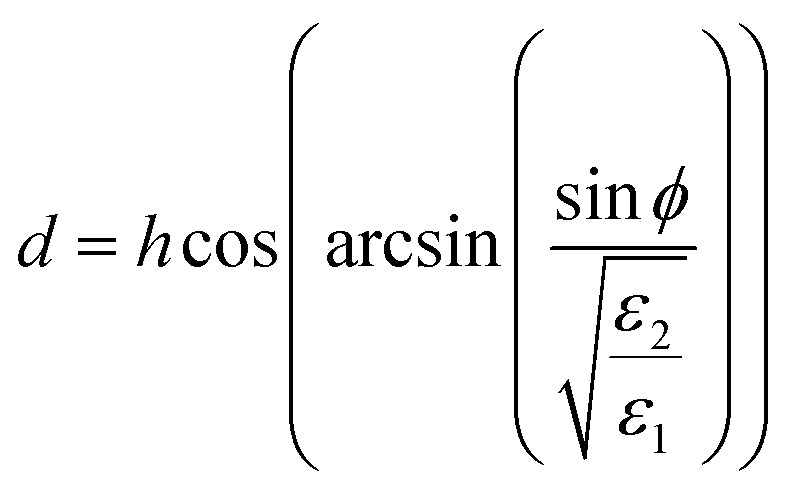
 is the thickness of medium-2 with *ε*_1_ and *ε*_2_ as the relative permittivity of medium-1 and 2, respectively and *ϕ* as the incident angle. The proposed model is passive in nature hence *S*_12_ = *S*_21_. Finally, the absorption is calculated as *A*(*ω*) = 1 − *R*(*ω*) − *T*(*ω*). Here, *T*(*ω*) is the transmission coefficient, which remains zero due to the presence of the ground plane. Fig. 4(c) compares the absorption spectra obtained from the simulation in the coupled system and calculated for the decoupled system using the multiple reflection theory. It can be seen in the absorption plot that the simulated spectra for the coupled system is in strong correlation with that calculated obtained using the interference theory validating the operation of the proposed absorber. Fig. 4(d) shows the profile of the accepted power from the applied input ports in medium-1 and power loss in absorber unit cell. This loss profile is obtained by the full wave simulation. It can be noticed in the power loss profile that the ground plane is being utilized as a reflecting plane only while the main absorption occurs due to the scattering characteristics of the slotted graphene-dielectric interface. This follows the concept of interreference theory confirming the cause of absorption as destructive interference occurring in medium-2. Fig. 4(d) also shows the surface power loss density confirming the power loss pattern at the frequency of the fundamental and higher order mode occurs in the graphene patch only. The surface power loss density remains approximately zero in the other parts of the absorber unit. Here, the obtained absorption plot is calculated with the normal incidence of the electromagnetic wave and it is also applicable for the oblique incidence.^35^

## Absorber performance analysis

**Fig. 5:**
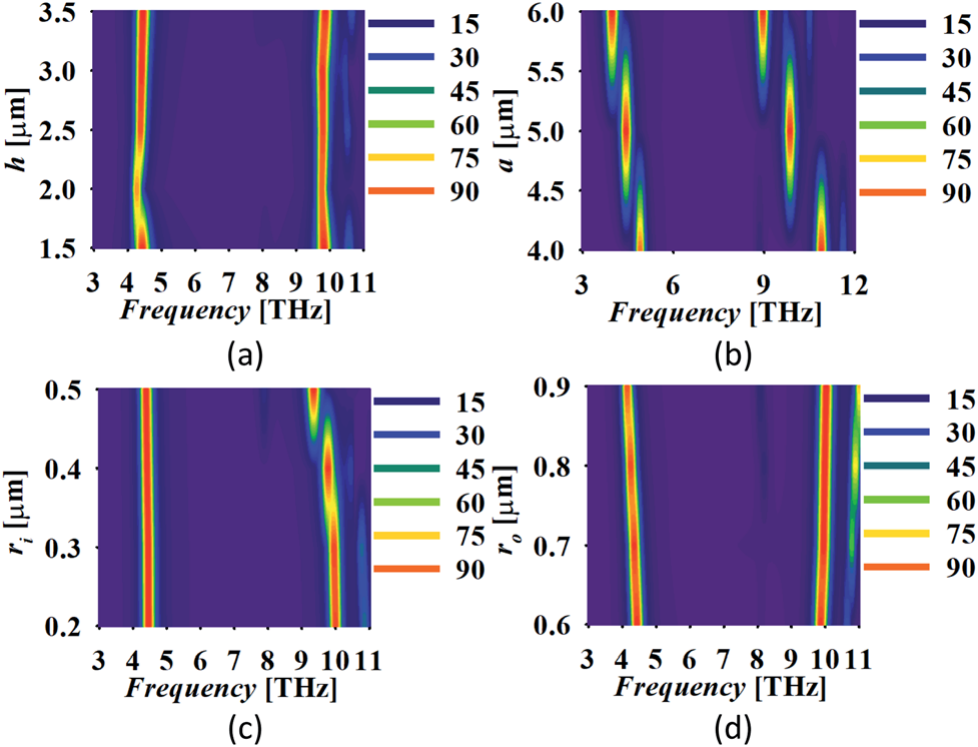
The absorber response with the variation in (a) *h*, (b) *a*, (c) *r*_i_ and (d) *r*_o_ (all the dimensions are in μm).

The absorber performance is analysed in terms of the variation in its physical parameters for the consideration of fabrication tolerances. One physical parameter is varied at a time and others are kept intact. The variation in the absorber response with the variation in substrate height *h*, the edge of the graphene patch, *a*, inner and outer radius of the inserted annular circular slot *r*_i_ and *r*_o_, respectively is shown in Fig. 5. The variation in *h* shows the perfect absorption in both the bands if its value is selected as *h* ≈ *λ*_sp_/4(*h* = 2.5 μm and above), as shown in Fig. 5(a). The minimum value of *h* is selected to maintain the ultrathin geometry. It can be noticed that a much smaller value of *h* can also be selected at the cost of nearly perfect absorption. There is a redshift in the response with a small shift in the lower and large shift in the upper band with the increment in *a* as shown in Fig. 5(b). Thus, the appropriate selection of *a* can allow setting of the operating frequency. The insertion of the annular slot affects the absorption at the frequency of the higher order mode only. Similar results can be observed in Fig. 5(c) which depicts the absorption spectra with the variation in *r*_i_. Fig. 5(d) represents that the increment in *r*_o_ separates the resonant bands with a larger gap with the reduced value of absorption in the lower band.

**Fig. 6:**
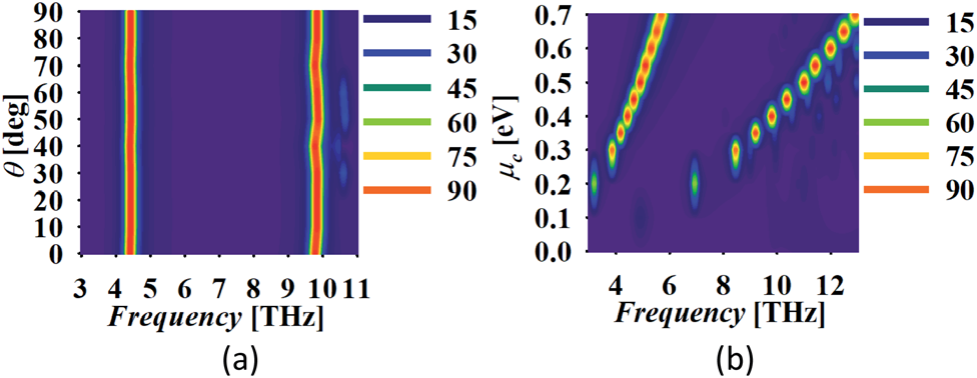
Absorber response with the variation in (a) *θ* and (b) *μ*_c_.

The symmetric structure of the absorber provides the insensitivity to the polarization angle. Moreover, Fig. 6(a) shows the absorption spectra with the variation in the angle of incidence of the electromagnetic wave, *θ*. The absorption spectrum is approximately stable over the variation in *θ* in both the operating bands with slight deviation in the peak frequency of the upper band. The insensitivity to the angle of incidence makes the proposed absorber capable of being utilized in non-stationary handheld devices which generally remains quite difficult to implement.

## Tuning of absorber response

Once the dimensions are set for the desired frequency operation, the structure can be fabricated and a further variation in frequency response can be governed by the variation in the chemical potential of graphene. The chemical potential of graphene can be varied by applying a DC bias voltage on the graphene patch. An ion-gel layer of thickness 0.1 μm with permittivity *ε*_ig_ = 1.82 is coated over the graphene patch so that all the periodically arranged unit cells can be biased together.^37,45,50,51^ The height of the substrate is reduced correspondingly to *h* = 2.43 μm so that resonant bands can be maintained at similar frequencies. Fig. 6(b) shows the absorption curve with the variation in the chemical potential of graphene, *μ*_c_. The increment in *μ*_c_ blueshifts the frequency response.^52^ There is a small and large shift in the lower and higher mode, respectively with the variation in *μ*_c_. Kubo's formalism can be applied for defining the conductivity of graphene material with the selection of parameters as relaxation time *τ* = 0.1 ps and temperature *T* = 300 K with the single layer of graphene with thickness as *t*_g_ = 0.34 nm.^6,53^ A number of research articles are available discussing graphene's conductivity, for more detail please consult ref. 54. Here, the graphene material available in CST library is utilized for the simulation by setting the parameters considering all the physical terms appropriately. It is necessary to note that the proposed geometry of the graphene patch placed on the dielectric substrate over a ground can behave as a perfect reflector in the lower THz regime for *μ*_c_ < 0.2 eV. Absorption peaks are obtained only when *μ*_c_ > 0.2 eV and the generated absorption peaks attain the value of more than 90% for *μ* > 0.3 eV.

## Sensing performance

### A. Variation in analyte thickness, *t*_a_

**Fig. 7:**
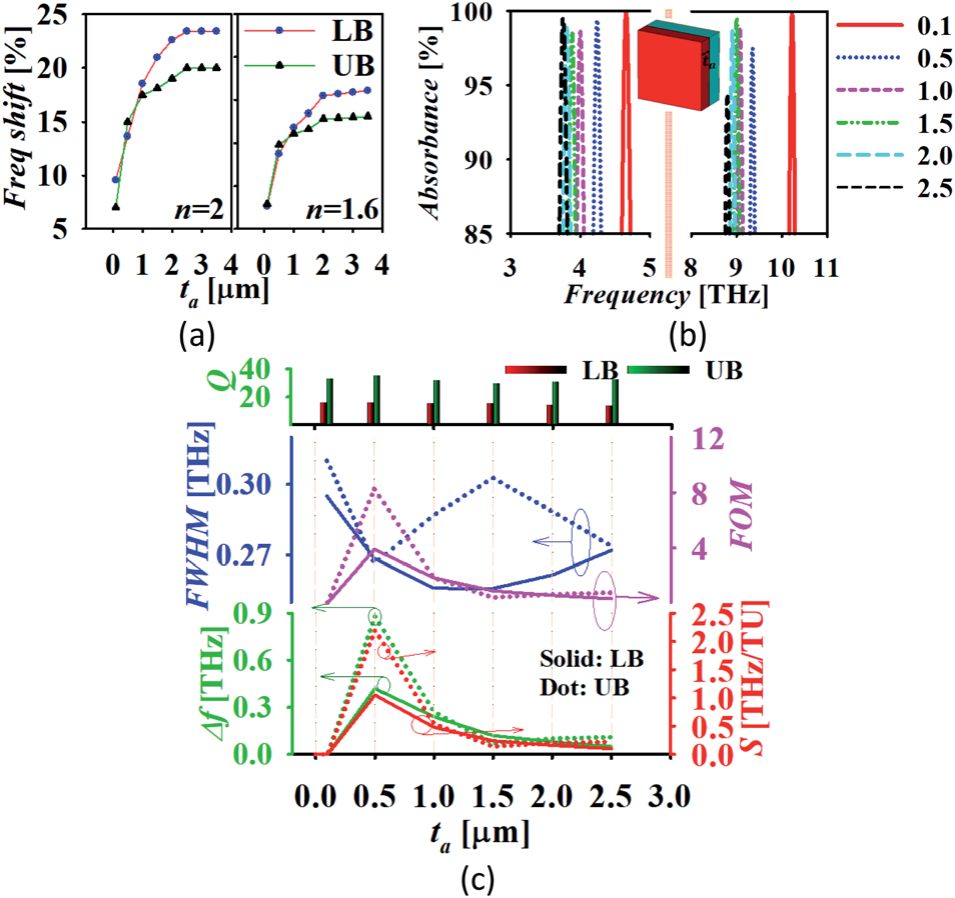
(a) The frequency shift, (b) the absorption spectrum with the variation in *t*_a_ and (c) absorption performance analysis (LB: lower band and UB: upper band).

Fig. 6(b) confirms that the best value of the chemical potential is *μ*_c_ = 0.5 eV for which the absorption peak touches the level of perfectness and hence can be selected for sensing applications keeping all other physical parameters similar as discussed earlier. For the analysis of the sensing performance, a layer of photoresist can be grown on the absorber and the effect of the fringing field is studied.^24,25^Fig. 7(a) shows a frequency shift in the absorption spectrum with the change in the analyte thickness for refractive index *n* = 2 and *n* = 1.6. The percentage of frequency shift (FS) is calculated using 
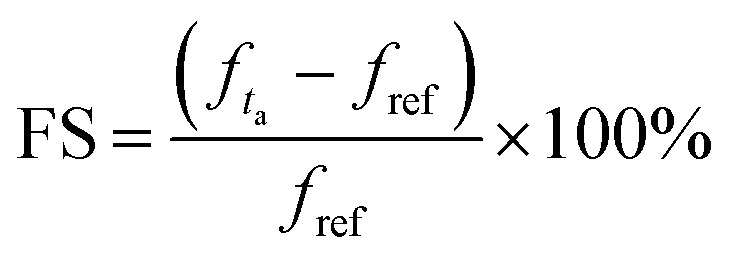
. Here, *ft*__a__ is peak absorption frequency with analyte thickness *t*_a_ and *f*_ref_ is the peak absorption frequency without analyte.^24^ The percentage change in the frequency shift is reduced at the lower value of refractive index when thickness is varied. Moreover, the frequency shifting is saturated at the value of *t*_a_ around 2 μm, which is selected for reporting the refractive index sensing performance of the proposed absorber in the next subsection.^24,25^Fig. 7(b) represents the absorption spectrum with the variation in *t*_a_ with the analyte of refractive index *n* = 2. Fig. 7(c) shows the absorber performance in terms of different parameters like sensitivity (*S*), full-width half-maxima (FWHM), figure-of-merit (FOM), quality-factor (*Q*) and frequency shift Δ*f* over the unit variation in the analyte thickness Δ*t*. Here, Δ*f* = *f*_2_ − *f*_1_, *f*_1_ is the initial frequency and *f*_2_ is the frequency after shifting when analyte thickness is varied from *t*_1_ to *t*_2_ and Δ*t* = *t*_1_ − *t*_2_.^17^ The performance parameters are calculated as 
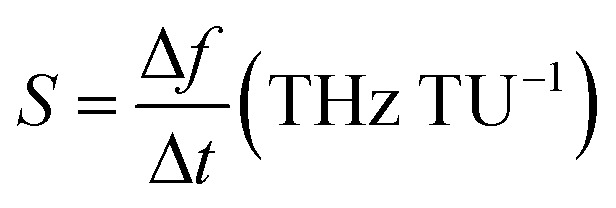
 (TU: thickness unit), 
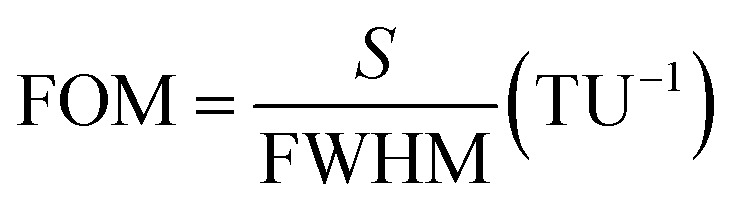
 and *Q* = *f*_p_/FWHM. Here, *f*_r_ is the peak absorption frequency. These parameters are calculated and plotted in Fig. 7(c) for varying *t*_a_. The proposed absorber provides high sensitivity, FOM and *Q*-factor at thickness *t*_a_ ≈ 0.5 μm. The highest sensitivity obtained with the variation in the analyte thickness is 1.05 THz TU^−1^ and 2.2 THz TU^−1^ in the lower and upper band, respectively. The sensitivity and FOM are significantly reduced for the higher values of *t*_a_. It shows that the proposed absorber can be suitably fit for the biosensing application because the thickness of most of the viruses remains within *t*_a_ < 1 μm.^8^

### B. Effect of the separation between the sensor and analyte

**Fig. 8:**
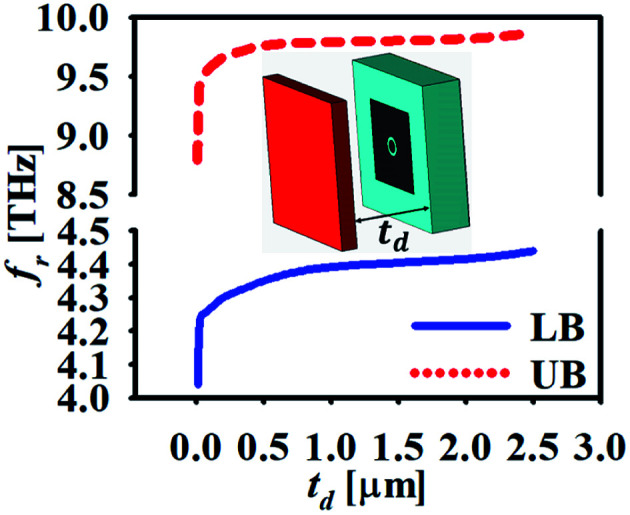
The variation of resonance peak frequencies for varying *t*_d_ maintaining the absorption of more than 90%.

As stated earlier, the proposed biosensor is independent of the incident angle and hence its capability must be checked for its usage in dynamic systems by varying the spacing between the analyte and sensor. Fig. 8 shows the plot of peak absorption frequency with the variation in distance of the analyte form sensor, *t*_d_. The resonant frequencies of both the operating bands respond in the same manner and there is drastic variation till the value of *t*_d_ = 0.5 μm. For *t*_d_ > 0.5 μm, the shift in the peak resonant frequencies is saturated over the variations in *t*_d_. In this exercise, the value of the refractive index of the analyte is selected as *n* = 1.2 with its thickness as *t*_a_ = 2 μm.

### C. Refractive index sensing

**Fig. 9:**
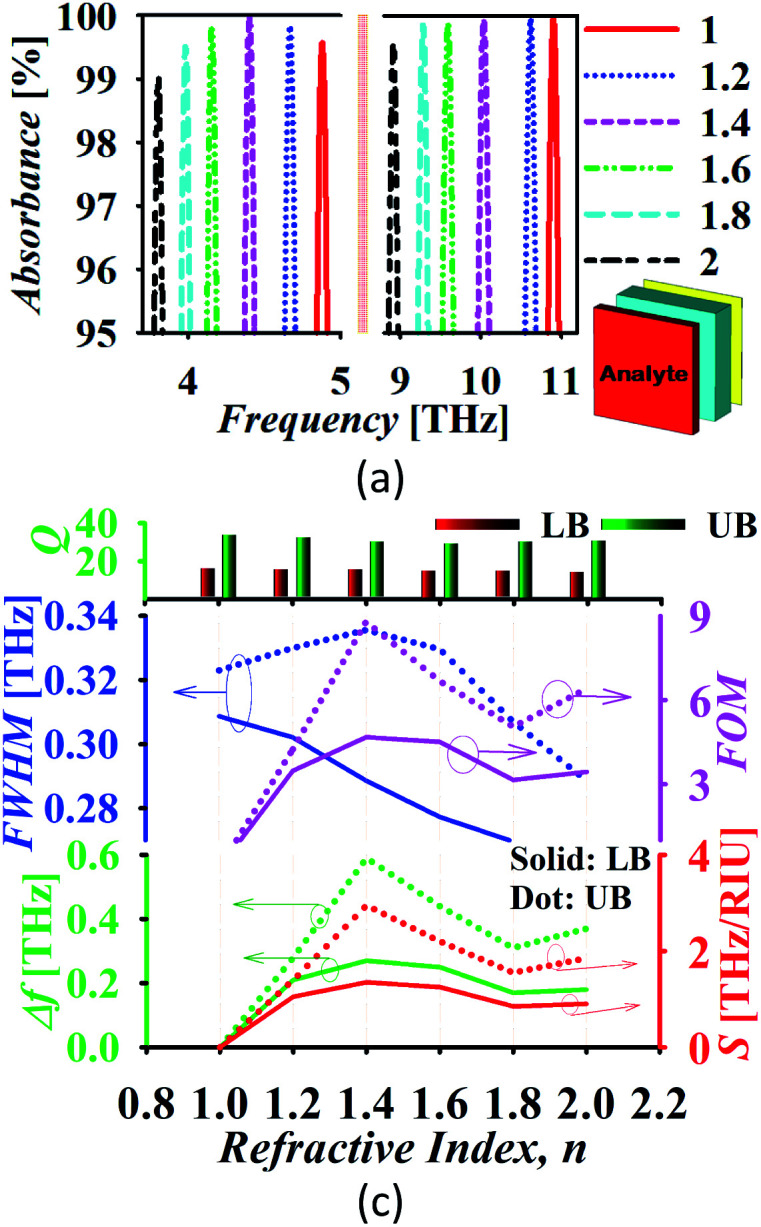
(a) The absorption spectrum during refractive index sensing and (b) absorber performance analysis (LB: lower band and UB: upper band).

For the utilization of the proposed absorber in refractive index sensing (RIS) applications, an analyte of thickness *t*_a_ = 2 μm is placed at the top of the absorber. Fig. 9(a) illustrates the absorption spectrum with the variation in refractive index and Fig. 9(b) shows the absorber performance parameters. Here, the frequency shift Δ*f* is considered over the unit variation in refractive index Δ*n*. Here, Δ*f* = *f*_2_ − *f*_1_, *f*_1_ is the initial frequency and *f*_2_ is the frequency after shifting when the refractive index of analyte is varied from *n*_1_ to *n*_2_ and Δ*n* = *n*_1_ − *n*_2_. The sensitivity is calculated as 
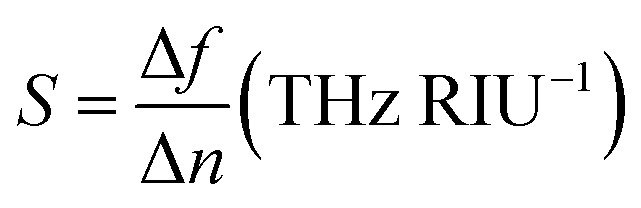
 (RIU-refractive index unit). It can be observed in Fig. 9(a) that there is a large shift in the peak frequency of the upper band in comparison to the lower band. Also, the sensitivity provided by the upper band is higher when the thickness of analyte is selected as 2 μm and the chemical potential of graphene as *μ*_c_ = 0.5 eV. In this scenario, the lower and upper band provides the maximum sensitivity of 1.35 and 2.95 THz RIU^−1^, respectively. The maximum FOM is 4.67 and 8.8 (RIU^−1^) for the lower and upper band, respectively. Moreover, the proposed absorber provides the narrow linewidth being defined by FWHM. The intensity of the absorption peak remains more than 99% over the variation in the refractive index. The proposed absorber can be utilized in the sensing of water (*n* = 1.333), ethanol (*n* = 1.36), ethylene glycol (*n* = 1.41), glycerine (*n* = 1.47), benzene (*n* = 1.5), sodium chloride (*n* = 1.54) and carbon disulphide (*n* = 1.63) with high sensitivity and other improved performance parameters in comparison to recently reported graphene based sensors.^55^

### D. Sensing with the variation in chemical potential

**Fig. 10:**
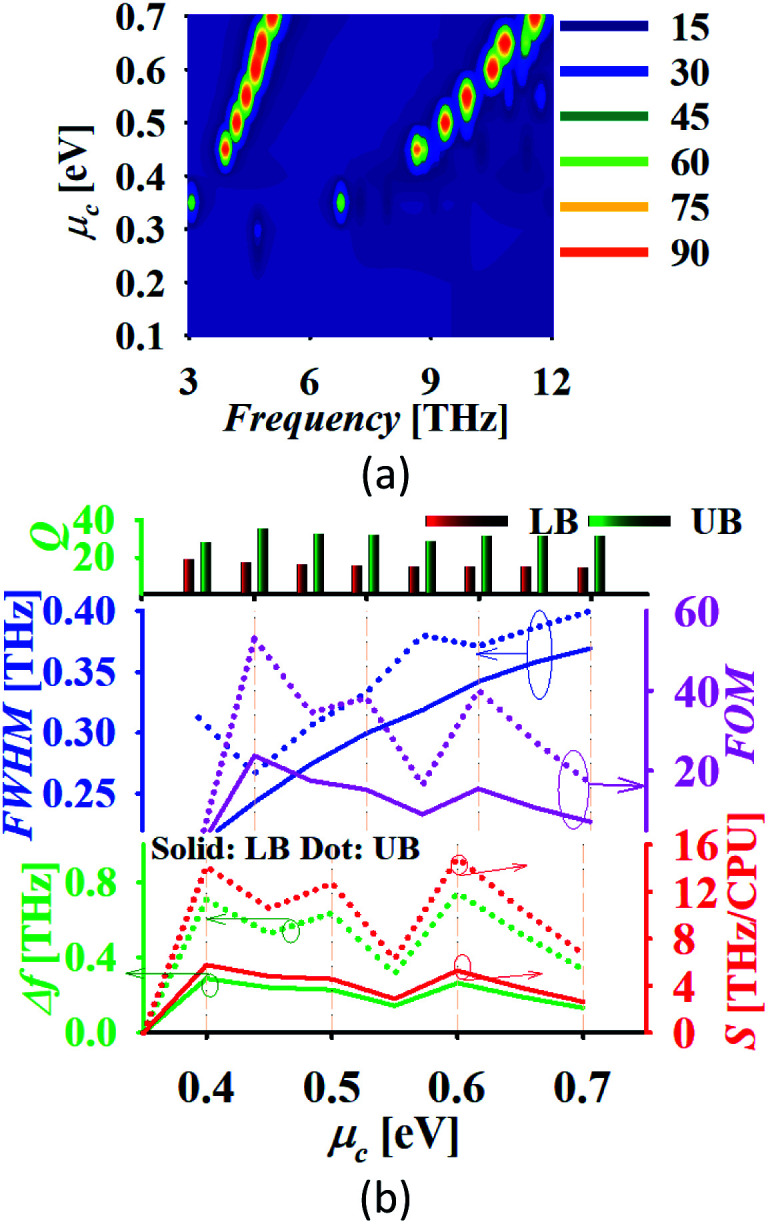
(a) Absorption tuning after placing an analyte of *t*_d_ = 2 μm and *n* = 1.2 and (b) absorber performance analysis.

An analyte with thickness 2 μm and refractive index, *n* = 1.2 is placed at the top of the sensor and the chemical potential of graphene is varied. Tuning of the absorption spectrum is possible with variation in *μ*_c_ as shown in Fig. 10(a). The analysis reported in Fig. 10(b) represents that the absorber response attains an ultra-high sensitivity of 5.76 and 14.88 THz CPU^−1^ (CPU: chemical potential unit) with a high value of FOM around 23.7 and 53.09 with the lower and upper band, respectively. The absorber shows the spreading in linewidth with the increment in *μ*_c_. Also, the absorber maintains a high *Q*-factor throughout the variation in *μ*_c_. The high sensitivity of the absorber over the variation in *μ*_c_ represents the high tunability and controllability of sensor.

## Detection of the malaria virus

Table 1 shows the performance of the proposed absorber when it is utilized in the detection of the malaria virus. The refractive index of red blood cells infected with different forms of malaria virus in trophozoite and schizont stages are 1.373 and 1.383, respectively.^8,56^ The proposed absorber provides high sensitivity and FOM, as expected. Here, the thickness of the analyte being considered as blood with malaria virus is taken as 2 μm with the electrical characteristics of graphene as *μ*_c_ = 0.5 eV and *τ* = 0.1 ps at *T* = 300 K.

**Table 1:** Performance during malaria virus sensing

*n*	LB		UB	
	*S* (THz RIU^−1^)	FOM (RIU^−1^)	*S* (THz RIU^−1^)	FOM (RIU^−1^)
1.373	1.29	4.40	2.34	6.79
1.383	1.34	4.64	2.43	7.10

## Glucose detection

**Fig. 11:**
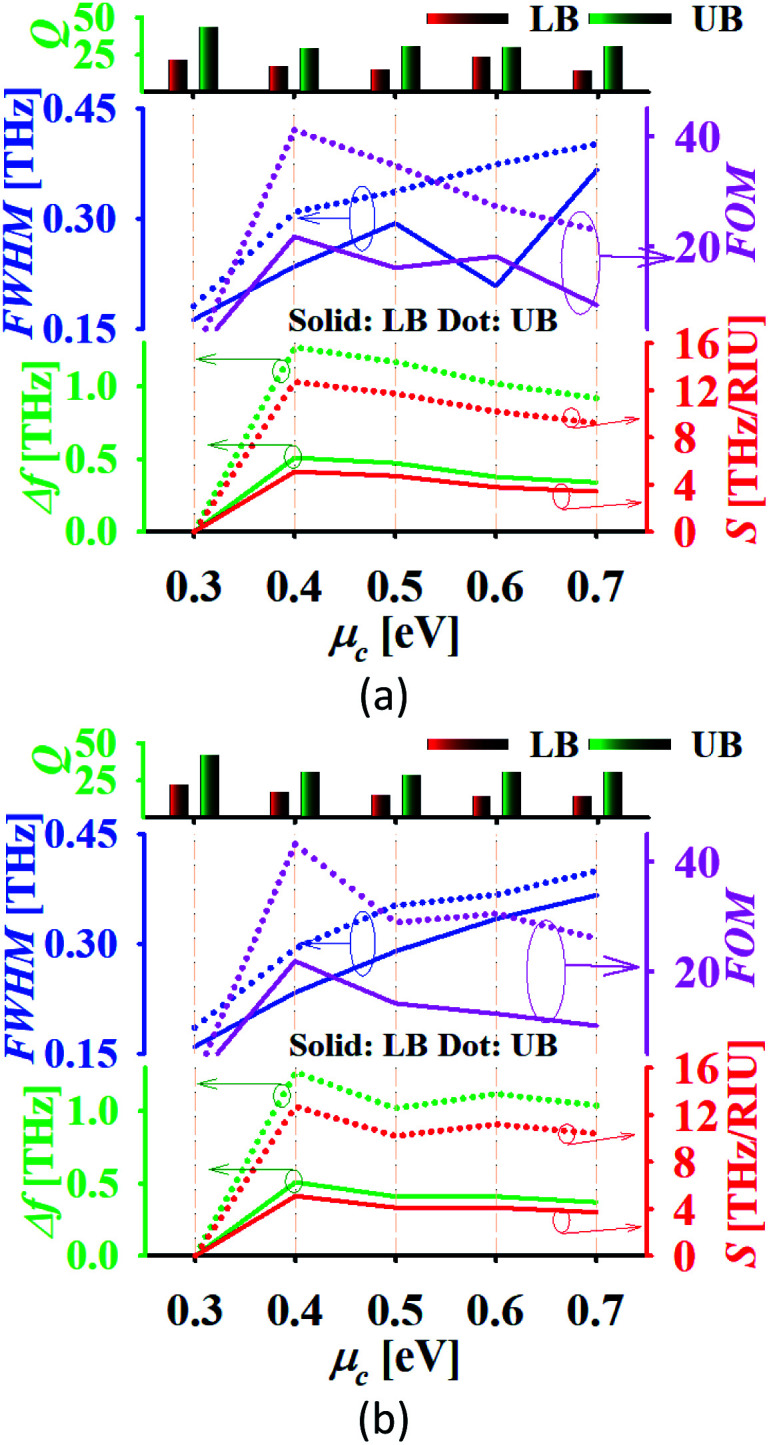
Performance of the dual-band absorber in (a) water and (b) water with 25% glucose.

The performance of the proposed dual-band absorber is analysed in the water with refractive index 1.3198 and in water with 25% glucose with refractive index 1.3594, as illustrated in Fig. 11.^8^ The plots represent that there is a slightly increased linewidth but the absorber still offers a high FOM due to the high sensitivity in both cases. The absorber offers the sensitivity 5.1 and 12.7 THz RIU^−1^ with FOM as 21.73 and 41.1 RIU^−1^ in the lower and upper band, respectively in the case of pure water for *μ*_c_ = 0.4 eV and *τ* = 0.1 ps. The absorber offers the maximum sensitivity in the case of 25% glucose mixed water similar as in water with a little higher FOM of 21.86 and 43.212 RIU^−1^ in the lower and upper band, respectively. The other variations in the glucose concentration in water can also be detected using the proposed absorber with satisfactory performance parameters.^57^

A comparative analysis is reported in Table 2. The proposed work is compared with three different operations; (a) as a refractive index sensor (RIS), (2) with the variation in the thickness of analyte used for sensing purposes and (3) with the variation in *μ*_c_. The proposed absorber offers better performance in terms of sensitivity, FOM and *Q*-factor in comparison to recently reported other sensors. Also, the proposed tunable absorber offers a thin geometry with strong angular and polarization stability in comparison to others. Recently, some metamaterial based biosensors have been fabricated and reported which show a strong agreement in the simulated and measured results.^24,25,58^ This recent literature supports the hope that the absorber/sensor reported in this article will perform as expected if experimentally implemented in the future.

**Table 2:** A comparison with other biosensors

Ref		Bands	*f* (THz)		*S* (THz RIU^−1^)		FOM (1/RIU)		*Q*		Thickness (um)	Polarization insensitivity	Tunability	Allowed incident angle
			LB	UB	LB	UB	LB	UB	LB	UB				
55		2	5.16	10.3	1.04	2.34		28.3			5.2	Yes	Yes	75° with 80% absorption
59		2	1.8	2.26	0.1875	0.360	7.2	19.1	120	94	8.6	Yes	No	30°
60		2	1.08	2.77	0.147	0.964	1.21	17.28	8.9	55	10.4	Yes	No	Not reported
61		2	0.5	0.94	—	—	—	—	—	—	10	No	No	Not reported
62		2	7.1	10.4							4.1	Yes	Yes	70°
63		2	1.42	2.99	0.28	1.48	1.2	24.6	7.11	59.8	10.8	Yes	No	Not reported
64		2	1.7780	2.4591		1.90		229	6.91	296	4.8	No	No	Not reported
65		2	0.76	1.28	0.47	0.51	9.4	14.4	23	3.7	5.4	Yes	No	Not reported
28		2	0.5	1.425	0.0001		—		—		642	No	No	Not reported
29		2	0.68	1.17	0.0367	0.0239	—	—	28	65	502	No	No	Not reported
24		1	0.6/0.75		0.007/0.00618		2.3/1.98		11.6/11		518.2	Yes	Yes	Not reported
25		1	0.4		0.0273		—		9.6		502	No	No	Not reported
43		1	0.86		0.006		0.2		4.5		25	No	No	Not reported
42		1	1		0.016/0.028		—		—		25	No	No	Not reported
41		1	0.6		0.016		—		6		500	No	No	Not reported
This work	RIS	2	4.88	10.9	1.35	2.95	4.67	8.8	15.25	29.88	2.52	Yes	Yes	Insensitive to *θ*
	*t*_a_				1.05	2.2	3.91	8.25	15.8	35.09				
	*μ*_c_				5.67	14.88	23.7	53.09	17.17	35.09				

## Conclusions

A graphene based tunable ultrathin narrow dual-band absorber has been numerically analyzed and implemented. Fundamental and third order surface plasmon modes have been generated in the absorber geometry which provide an absorption of more than 99% and 50% in the lower and upper band, respectively. The technique has been implemented for electrostatic charge trapping and creation of the bipolar charged nodes on the graphene air interface by inserting an annular slot of dimensions equivalent to *λ*_sp_/2. Consequently, the absorption in the upper band has been enhanced to more than 99%. The operation of the proposed absorber has been verified by interference theory confirming the cause of absorption as destructive interference in the dielectric being used as the substrate. The frequency response of the absorber remains insensitive to polarization and incidence angle. The performance of the proposed dual-band absorber has been analyzed as a refractive index sensor and different biomedical applications like the detection of glucose in water and detection of malaria virus in the blood. An ultrahigh sensitivity of 14.88 and 12.7 THz RIU^−1^ with FOM as 54 and 41.1 RIU^−1^ at the frequency of upper band has been obtained. The sensing performance in the upper band remains better in comparison to the lower band. The proposed absorber can potentially be utilized in dynamic hand held devices.

## Conflicts of interest

There are no conflicts to declare.

## Supplementary Material
